# Immobilization precision of a modified GTC frame

**DOI:** 10.1120/jacmp.v13i3.3690

**Published:** 2012-05-10

**Authors:** Brian Winey, Juliane Daartz, Frank Dankers, Marc Bussière

**Affiliations:** ^1^ Department of Radiation Oncology Massachusetts General Hospital Boston MA; ^2^ Harvard Medical School Boston MA; ^3^ Department of Radiation Oncology Paul Scherrer Institute Villigen CH‐5232 Switzerland; ^4^ Department of Applied Physics Eindhoven University of Technology Eindhoven The Netherlands

**Keywords:** stereotactic radiosurgery, immobilization, patient setup

## Abstract

The purpose of this study was to evaluate and quantify the interfraction reproducibility and intrafraction immobilization precision of a modified GTC frame. The error of the patient alignment and imaging systems were measured using a cranial skull phantom, with simulated, predetermined shifts. The kV setup images were acquired with a room‐mounted set of kV sources and panels. Calculated translations and rotations provided by the computer alignment software relying upon three implanted fiducials were compared to the known shifts, and the accuracy of the imaging and positioning systems was calculated. Orthogonal kV setup images for 45 proton SRT patients and 1002 fractions (average 22.3 fractions/patient) were analyzed for interfraction and intrafraction immobilization precision using a modified GTC frame. The modified frame employs a radiotransparent carbon cup and molded pillow to allow for more treatment angles from posterior directions for cranial lesions. Patients and the phantom were aligned with three 1.5 mm stainless steel fiducials implanted into the skull. The accuracy and variance of the patient positioning and imaging systems were measured to be 0.10±0.06 mm, with the maximum uncertainty of rotation being ±0.07°.957 pairs of interfraction image sets and 974 intrafraction image sets were analyzed. 3D translations and rotations were recorded. The 3D vector interfraction setup reproducibility was 0.13 mm ±1.8 mm for translations and the largest uncertainty of ±1.07° for rotations. The intrafraction immobilization efficacy was 0.19 mm ±0.66 mm for translations and the largest uncertainty of ±0.50° for rotations. The modified GTC frame provides reproducible setup and effective intrafraction immobilization, while allowing for the complete range of entrance angles from the posterior direction.

PACS number: 87.53.Ly, 87.55.Qr

## I. INTRODUCTION

Regardless of the treatment modality (Gamma Knife, CyberKnife, linac‐based, Proton, image‐guided or non‐image–guided), the goals of immobilization for stereotactic treatment include: (a) no intrafractional motion, (b) reproducibility over multiple fractions for fractionated cases, (c) limited interference with treatment beams, (d) no added imaging artifacts at the simulation or during treatment, and (e) patient comfort which benefits (a) and (b). Until recently, the standard stereotactic immobilization methods for single‐fraction treatments employed invasive fixation devices using pins placed against the patient's skull bones. While providing excellent intrafractional immobilization,^(^ ^1^ ^)^ the limits of invasive frames can include fewer fractionation options, patient discomfort, risk of frame slippage, and tight time constraints on treatment days for simulation, planning, and quality assurance.

Noninvasive frames have been employed for large fraction treatments.^(^ ^2^ ^,^ ^3^ ^)^ Recently, noninvasive frames have become more popular for immobilization of single‐fraction patients, often in an image‐guided framework.(3–11) Noninvasive frames either employ a bite block and posterior head support, or a thermoplastic mask with either a second posterior thermoplastic support or posterior cup. Some thermoplastic mask designs also include an upper palate fixation mechanism.^(^ ^5^ ^,^ ^10^ ^)^ There are several studies which have analyzed the precision and reproducibility of both types of noninvasive fixation devices^(^ ^5^ ^,^ ^11^ ^–^ ^13^ ^)^ and performed comparisons of the invasive frames to noninvasive methods.^(^ ^8^ ^,^ ^10^ ^)^ In our stereotactic clinic, we have designed a modified bite block frame based upon a commercially available noninvasive Gill‐Thomas‐Cosman relocatable frame^(^ ^2^ ^)^ system (Integra‐Radionics, Burlington, MA), and have used it to treat over 1200 patients, both single‐fraction and fractionated stereotactic patients.

The primary reasons for our design and modification of the commercially available GTC frame are the inclusion of a radiotransparent material of the posterior head support for greater beam angle access, and a larger posterior cup for greater reproducibility and less intrafraction motion. Additionally, less image guidance is required when a high‐precision frame is employed. It is for this reason that we seek to determine the precision of our mGTC frame. We present a retrospective study of image‐guided SRT patients immobilized with the modified GTC frame, including interfraction and intrafraction precision alongside a phantom‐based test of our imaging system accuracy and precision.

## II. MATERIALS AND METHODS

### A. mGTC frame

**Figure 1:**
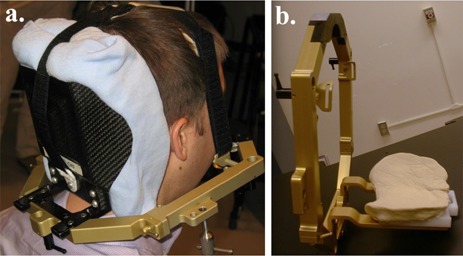
The author in a custom mGTC frame (a). The black carbon cup and the blue patient‐specific pillow can be seen in the posterior support of the frame. The Velcro straps and bite block of the original GTC frame are in place. A view of the original GTC frame (b) with the metal supports, plastic base and moldable plastic occipital cushion.

The mGTC is a “modified” adaptation of Integra‐Radionics' GTC relocatable stereotactic frame. Because of the material composition of the commercial occipital accessories, posterior lesions are not ideally suited for X‐rays and are inappropriate for protons. These accessories include two aluminum braces and a stepped plastic base which supports dense molding putty (Reprosil, Dentsply International, York, PA). The first implementation of the mGTC was in 2002, with the goal of using a noninvasive fixation for image‐based proton radiosurgery. The current mGTC design incorporates several improvements over the original model (Fig. 1), and is employed in both the proton and photon facilities of our clinic.

The commercial occipital cup is replaced by a 1/32 inch thin carbon concave support surface, which surrounds the base of skull. The plate is shaped to securely mate with a patient‐specific cushion (Moldcare, ALCARE Co. Ltd., Tokyo, Japan), which makes contact with the patient's head. An extended bit mold mount ensures the carbon cub sits more inferiorly compared to the conventional device. The shape and length of the improved materials provide good immobilization, starting at the upper neck and including the head.

The modifications have the effect of limiting density gradients across the immobilization device, which is needed to minimize range uncertainties for protons, providing more uniform background for high‐quality imaging and reducing attenuation for photons.

### B. Retrospective patient study

**Figure 2:**
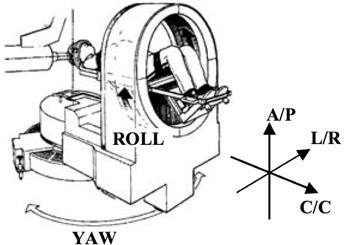
The STAR patient positioning system. The two orthogonal kV imaging systems are not shown in this image, but are found along the beamline and orthogonal to the beamline.

All patients included in this retrospective study were treated on the STereotactic Alignment for Radiosurgery (STAR) system located at the FH Burr Proton Therapy Center at Massachusetts General Hospital (Fig. 2). A retrospective IRB was approved for this study. STAR is a static proton beam line with a patient positioning system capable of 0.05 mm precision translations and 0.05° precision rotations. It is a five‐degree‐of‐freedom system, with three linear translations and two rotations, roll about the cranial–caudal axis and yaw about the anterior–posterior axis. The positioning system accounts for pitch about the left–right axis by utilizing a rotation of the proton beam aperture when treating with lateral fields. The STAR system incorporates a pair of orthogonal room‐fixed kV imaging sources, and imaging panels located along and orthogonal to the beam line.

Patients undergoing proton stereotactic treatment are fitted with a custom mGTC frame. Additionally, three 1/16 inch diameter surgical steel ball fiducials are implanted into the outer shelf of the patients' skulls prior to the initial planning CT acquisition. The fiducials are identified in the treatment planning system, and coordinates of the fiducials and isocenter(s) are transferred to the patient treatment imaging system. The patient treatment imaging systems requires fiducials to perform a for six‐degree‐of‐freedom alignment using a simple ray tracing back‐projection algorithm, as it is incapable of performing an intensity‐based 2D/3D alignment using digitally reconstructed radiographs (DRRs).

Since the STAR system employs a fixed proton beam line, posterior and anterior beam directions require rolling the patient onto their side. Since most institutions do not roll patients 90° onto their sides, this retrospective study selectively excludes image pairs of rolled treatment fields for intrafractional motion analysis.

### C. Setup verification

A software package, developed in‐house, has been deployed for patient setup verification (DIPS). The software package allows for the import of digitally reconstructed radiographs (DRRs) from the treatment planning software, along with field apertures, critical structures, and points of interest. The DRRs and points of interest are stored in the database as volumetric data and displayed in the GUI as planar points. The implanted fiducials and isocenter(s) are labeled in the treatment planning system as points of interest and transferred to the DIPS database.

On the day of treatment, the software package retrieves the patient‐specific isocenter and fiducial locations from the database, and projects the isocenter and fiducial locations onto the DRRs for reference. An initial pair of orthogonal kV images is acquired, and the centers of the fiducial shadows are selected on each of the orthogonal kV images. The DIPS software performs a ray casting back projection to determine the room position of the fiducials, and then performs a least squared distance optimization in six degrees of freedom to determine the patient shift. The process is repeated prior to all treatment fields unless one of imaging directions is blocked by the patient (i.e., superior fields); then only two translations and one rotation are measured.

### D. DIPS and STAR accuracy and precision

The DIPS imaging system and STAR positioning system have been tested with a cranial phantom to ascertain the accuracy and precision of the combined patient positioning and imaging systems. The tests employed a cranial phantom with three implanted fiducials and 45 predetermined shifts were introduced, including translations, rotations, and combinations of both. The DIPS imaging software system was used to measure the translations and rotations, and compare them to the physical shift. The accuracy was calculated as the difference between the shift applied by the positioning system and the feedback of the fiducial based imaging system and the precision was the variance of the measurements. Direct measurements of the STAR patient position system were performed using linear encoders. It is important to note that the accuracy of the image registration software and patient positioning system was measured independently of a full end‐to‐end accuracy test; the accuracy of the DIPS imaging system and patient positioner do not include errors of CT delineation, treatment planning, patient motion or imaging/radiation isocenter coincidence.

### E. Analysis

Forty‐five fractionated stereotactic patients treated since September 2009 were retrospectively analyzed for this study with a total of 1002 fractions, 957 interfraction image sets, and 974 intrafraction image sets. Due to the treatment room geometry, some of the intrafraction image sets include only one image due to a collision risk of the orthogonal imaging panel and, thus, only three dimensions were included for the analysis (two planar dimensions and one rotational dimension). For all other images sets, all six degrees of motion were analyzed.

Interfraction precision of the mGTC was measured using the first day's setup position as the baseline reference position and all other images sets for subsequent days are relative to the initial setup. An end‐to‐end accuracy study was not included in this study; therefore, all statements of patient position are relative and only define the precision of the image guidance. Intrafraction motion was assessed as the motion relative to the first field treated in a given fraction. All interfraction and intrafraction shifts above 0.2 mm and 0.2° were applied to the patient position. As stated above, only patients and fractions including nonrolled fields were included in the analysis, for more accurate comparison to nonfixed beam patient treatment scenarios. Rolling a patient up to 90° can introduce large intrafraction motions, and few institutions employ fixed‐beam stereotactic treatments.

## III. RESULTS

### A. DIPS and STAR accuracy

Forty‐five combinations of translations and rotations ranging from −3mm to 3mm and −3° to 3° for all six axes were simulated using a cranial phantom. Three (3) mm and 3° were picked as being representative of the largest interfractional shifts we routinely observe in the clinical setting. Some patients require larger shifts, but repeat imaging is required according to our clinical protocol when large shifts are suggested. Since the STAR system cannot produce a pitch rotation about the left–right axis, a precision machined wedge of 3°, which can be used for treatments, was introduced to the phantom attachment to simulate a patient pitch.

**Figure 3:**
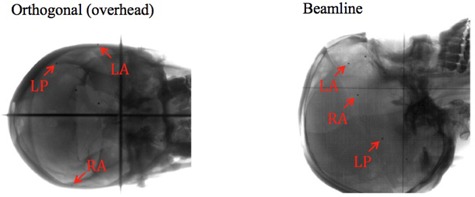
The cranial phantom as imaged with the kV imaging systems. The three fiducials are visualized and labeled on both images. The beamline image is on the right and the orthogonal (overhead) image is on the left. The cranial phantom contains other implanted markers not employed in this study.

The kV X‐ray image of the cranial phantom is seen in Fig. 3. The fiducials were manually selected in the DIPS system just as in a real patient treatment. The shifts measured by the DIPS system were compared to the predetermined shifts introduced by the STAR positioning system, and the difference was taken as the accuracy of the imaging and patient positioning systems. Table 1 summarizes the combined DIPS/STAR system accuracy and error when compared to the predetermined shifts. The vector sum of the translational error was 0.10±0.06 mm (mean ± 1 standard deviation). No rotational error was greater than 0.07°.

**Table 1:** Summary of the average and the standard deviations of the differences for the translations and rotations reported by DIPS compared to the physically applied shifts and rotations.

*Dimension*	*Average (mm,°)*	*Vector Summation*
Lateral (LAT)	0.00±0.04	
Anterior/Posterior (AP)	−0.01±0.07	0.10±0.06 mm
Cranial/Caudal (CC)	0.06±0.05	
Pitch (about LAT)	−0.03±0.05	
Yaw (about AP)	−0.01±0.05	
Roll (about CC)	0.02±0.07	

### B. Retrospective patient analysis

A MATLAB (MathWorks, Natick, MA) script was written to survey the daily treatment logs of the DIPS database, which records the patient position after the performance of every kV image analysis. Table 2 summarizes the intrafraction and interfraction precisions of the 45 patients. The interfractional precisions for the six degrees of freedom are listed on the right column of the table and intrafractional precision on the left. All precision data are presented in the form: mean ± 1 standard deviation.

**Table 2:** Summary of the average offsets and the standard deviations for the translations and rotations after alignment with fiducials.

*Dimension*	*Intrafraction (mm,°)*	*Interfraction (mm,°)*
Lateral (LAT)	−0.12±0.37	−0.04±0.55
Anterior/Posterior (AP)	−0.09±0.37	0.09±1.29
Cranial/Caudal (CC)	0.11±0.41	0.09±1.13
3D Vector	0.19±0.66	0.13±1.80
Pitch (about LAT)	0.14±0.20	0.07±1.07
Yaw (about AP)	0.10±0.50	−0.08±0.51
Roll (about CC)	0.06±0.25	0.05±0.59

Interfractional and intrafractional precisions of the mGTC frame are all close to 0.1 mm and 0.1°, which is the error of the imaging and patient position system reported above. The vector sum of the interfractional setup precision has a standard deviation of ±1.80 mm versus ±0.66 mm for the intrafractional precision. No intrafractional precision measurements were above 0.5 mm or 0.5°, but the standard deviations of the interfractional precisions were greater than 1 mm/1° for three of the axes. It has been our experience that the bite block is most susceptible to interfractional pitch and CC/AP reproducibility, and the data in Table 2 displays this statistical uncertainty.

**Figure 4:**
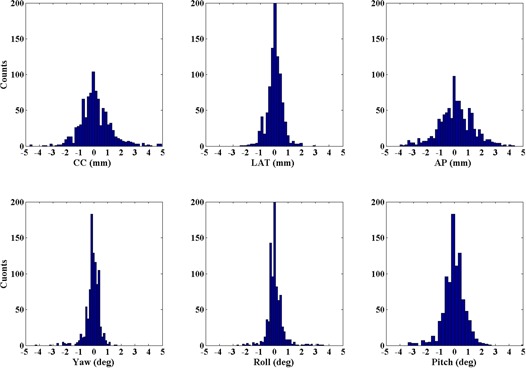
Histogram of the interfraction motions adjustments.

**Figure 5:**
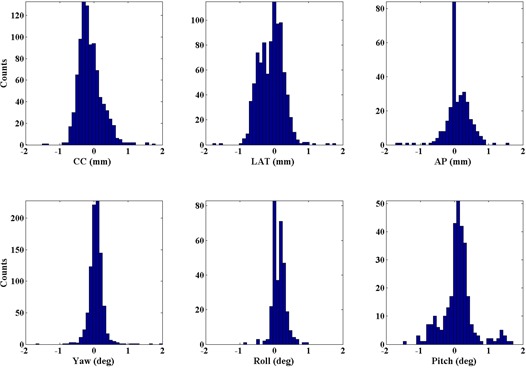
Histogram of the intrafraction motions. The AP, roll and pitch have fewer image sets due to the collision risk of the kV panel with the patient for some beam angles.

Figures 4 and 5 demonstrate the histograms for all images analyzed for interfraction and intrafraction displacements, respectively. Notice the difference in axes limits between the two figures with respect to the range of values, and within each figure with respect to the height of the histogram bins. The maximum intrafractional displacement is 1.8 mm for all patients. The AP, CC, and pitch interfraction histograms have the largest spreads, reflecting the larger values for σ reported in Table 2. As stated above, all interfraction and intrafraction shifts above 0.2 mm and 0.2° were applied to the patient position.

## IV. DISCUSSION

The mGTC frame developed at the Massachusetts General Hospital has been determined to provide precise immobilization similar to or better than other commercially available immobilization devices^(^ ^4^ ^,^ ^7^ ^,^ ^11^ ^,^ ^13^ ^)^ and nearly as precise as the invasive frame for intrafractional immobilization efficacy.^(^ ^8^ ^,^ ^10^ ^)^ Interfractional reproducibility is not as important as intrafractional when utilizing accurate and precise image‐guidance systems capable of accurately correcting interfractional shifts. Before the prevalence of image‐guidance systems, the precision of an invasive frame provided confidence of geometrically accurate patient treatment. The use of noninvasive immobilizations has allowed for more fractionation freedom and, combined with image guidance, accurate and precise daily treatments.

## V. CONCLUSIONS

This study looked at a modified GTC frame incorporating a novel occipital carbon cup and radiotransparent cushion. The modifications increased our confidence in the precision of the immobilization while decreasing the attenuation of the posterior support for posterior treatment angles. It has been shown that the mGTC frame, combined with implanted fiducials and an image‐guidance system, is capable of sub‐millimeter precision (±0.66 mm) during the treatment fraction, and no rotational uncertainty greater than ± 0.5°.
